# Asymptomatic giant retroperitoneal mass detected at a medical checkup

**DOI:** 10.1002/ccr3.1231

**Published:** 2017-10-16

**Authors:** Tsutomu Takeda, Daisuke Asaoka, Yuki Fukumura, Sumio Watanabe

**Affiliations:** ^1^ Department of Gastroenterology Juntendo University school of Medicine Tokyo Japan; ^2^ Department of Human Pathology Juntendo University School of Medicine Tokyo Japan

**Keywords:** Leiomyosarcoma, metastasis, retroperitoneal mass

## Abstract

The differential diagnosis of retroperitoneal mass includes liposarcoma, leiomyosarcoma, malignant fibrous histiocytoma, neurofibroma, stromal tumor, teratoma, and lymphoma. Leiomyosarcoma is rare with poorer prognosis than other soft tissue sarcomas. Soft tissue sarcoma of retroperitoneal origin often remains asymptomatic until tumor enlargement, leading to diagnosis at advanced stages.

## Question

**Figure 1:**
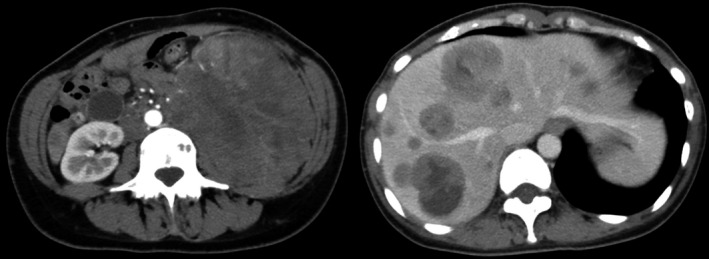
Computed tomography showed a solid tumor with irregular density in the left retroperitoneum and multiple liver metastases.

**Figure 2:**
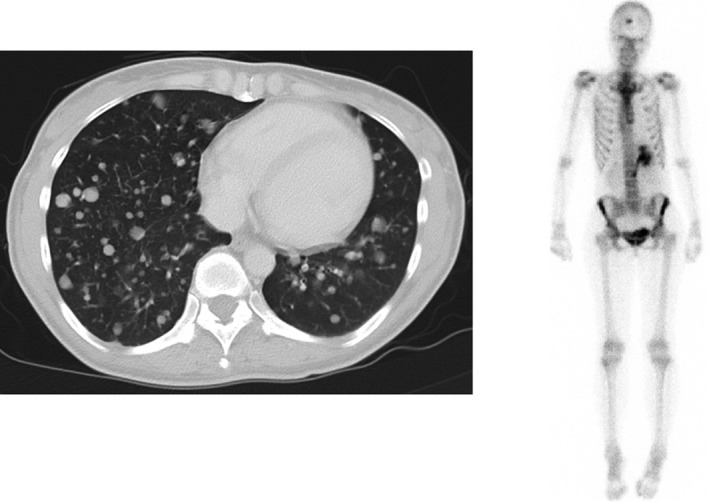
Computed tomography and bone scintigraphy showed multiple lung and bone metastases.

Multiple lung tumors were detected at a routine medical checkup at the office on a chest X‐ray in a 46‐year‐old woman. CT scanning identified a 150‐mm solid tumor with irregular density and some areas of weak enhancement in the left retroperitoneum, and multiple liver/lung/bone metastases (Figs 1 and 2). What is the clinical diagnosis?

## Answer

**Figure 3:**
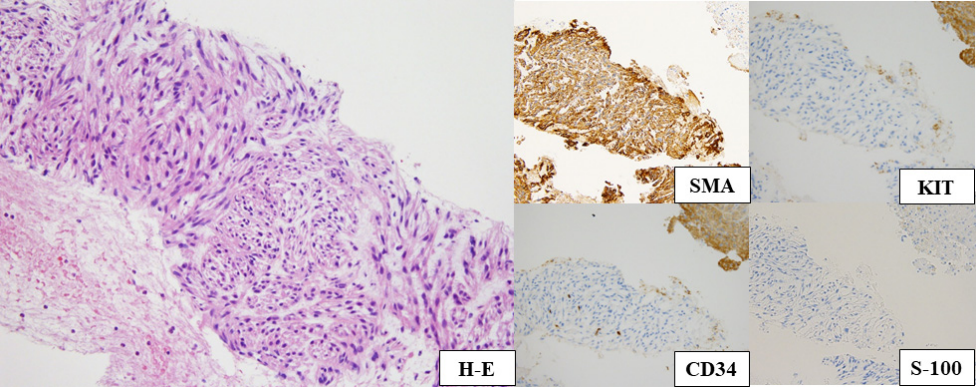
Medium power view of the H&E section showed atypical spindle cells, which were positive for smooth muscle actin (SMA) and negative for KIT, CD34, and S‐100 immunohistochemically.

A diagnosis of leiomyosarcoma was given based on a liver biopsy showing atypical spindle cells, which were positive for smooth muscle actin (SMA) and negative for KIT, CD34, and S‐100 immunohistochemically (Fig. 3). Leiomyosarcoma is a rare tumor with poor prognosis. Leiomyosarcoma of retroperitoneal origin often remains asymptomatic until tumor enlargement 1, 2. Most of the solid retroperitoneal neoplasms are of mesodermal origin, with liposarcomas, leiomyosarcomas, and malignant fibrous histiocytomas. Other differential diagnoses are neurogenic tumors, germ cell, sex cord, and stromal tumors and lymphoid and hematologic tumors such as neurofibromas, teratomas, and lymphomas 3, 4.

## Conflict of Interest

The authors state that they have no conflict of interest.

## Authorship

TT: gastroenterologist, responsible for the hospitalization and outpatient follow‐up of the case, and also had the major role in writing the manuscript. DA: gastroenterologist, responsible for the hospitalization and outpatient follow‐up of the case. YF: pathologist, responsible for pathological examination. SW: Professor of Department of Gastroenterology, Juntendo University School of Medicine; had the whole supervision of the case.
